# 

**Published:** 1917-07-14

**Authors:** 


### CONTENTS.

PAGE

**The Consumption of Beer** ... ... ... ... ... 285

**A War Emergency Benevolent Fund** ... ... 286

**Hospital and Institutional News** ... ... ... 287

A Director of Neurasthenic Institutions—Air-Raid Precautions at Guy's—Sir Victor Horsley and Colonel Carter—The Rutherfords and Newcastle Infirmary—The Mother of Children's Hospitals—Royal Infirmary, Liverpool—A Doctor's Educational Garden—Ontario Hospital, Orpington—A Hospital Chairman's Birthday—His Majesty as Almoner—"Doctor" an International Word—Brookfield Red Cross Hospital—Infirmary Medical Service in War Time—The Luck of the Mascot—The Doncaster New Infirmary—A Vast Extension Scheme—Officers and Their Food—The Abram Peel Hospital—Meningitis in New York—Coroner's Tribute to St. Bartholomew's Hospital—An Electrical Installation for Mansfield—German Feeding-Cups for the Red Cross—The Palais de Glace—Ashton Infirmary and the Explosion—Ashton Hospital Workers' Thanks—Goats for Institution Milk—Sphagnum Moss—An Infirmary Fire—The Salaries of Medical Women—This Week's Drug Market—To Our Readers.

**The Select Committee on the Medical Examination of Recruits** ... ... ... 293

Some Notes on the Evidence.

**How to Raise Birth-Rates After the War** ... 294

Interesting Figures and Sober Facts.

PAGE

**King George's Fund for Sailors** ... ... ... 295

Every Landsman's Duty and Opportunity.
Help for Our Sailors: The Mansion House Meeting.

**Guy's Hospital** ... ... ... ... ... ... ... 297

Twenty Years' Progress of a Great Charity.

**Hospital and Institutional Results** ... ... 298

**The Matrons' and Sisters' Department** ... 299

The Work of Hospital Almoners.

**The Uniform of the British Nurse** ... ... 300

Opinions of Leading Authorities—A Novel Change Suggested.
Round the Hospitals.
The Central Committee's Policy of Suicide—College Meeting at Whitechapel Infirmary—Fresh Fruit Teas for Nurses.

**Baby Week Conferences at Westminster** ... 302

The Problem of the Midwife.
The Illegitimate Child.

**Bird Life in Macedonia** ... ... ... ... 303

**Legal Notes for Institutional Workers** ... 304

**Matrons' and Sisters' Appointments** ... iv

**Parliament and the Profession** ... ... ... iv

**Passing Events and Topics...** ... ... ... iv

**The Institutional Worker** ... ... (Suppl.) 1

Electro-Medical Apparatus: Their Use, Construction, and Care.
The K.K. Empire Association.

